# Intrinsic Allergenicity Potential of Salt-Soluble Protein Extracts from the Diploid, Tetraploid and Hexaploid Wheats: Validation Using an Adjuvant-Free Mouse Model

**DOI:** 10.3390/ijms24065453

**Published:** 2023-03-13

**Authors:** Haoran Gao, Rick Jorgensen, Rajsri Raghunath, Shivam Chandra, Aqilah Othman, Eric Olson, Perry K. W. Ng, Venu Gangur

**Affiliations:** 1 Food Allergy and Immunology Laboratory, Department of Food Science and Human Nutrition, Michigan State University, East Lansing, MI 48824, USA; gaohaora@msu.edu (H.G.); jorgen70@msu.edu (R.J.); raghun14@msu.edu (R.R.); chandr77@msu.edu (S.C.); othmann1@msu.edu (A.O.); 2 Wheat Breeding & Genetics Laboratory, Department of Plant, Soil and Microbial Sciences, Michigan State University, East Lansing, MI 48824, USA; eolson@msu.edu; 3 Cereal Science Laboratory, Department of Food Science & Human Nutrition, Michigan State University, East Lansing, MI 48824, USA; ngp@msu.edu

**Keywords:** wheat allergy, intrinsic allergenicity, mouse model, anaphylaxis, IgE

## Abstract

Wheat allergies are potentially life-threatening and, therefore, have become a major health concern at the global level. It is largely unknown at present whether genetic variation in allergenicity potential exists among hexaploid, tetraploid and diploid wheat species. Such information is critical in establishing a baseline allergenicity map to inform breeding efforts to identify hyper-, hypo- and non-allergenic varieties. We recently reported a novel mouse model of intrinsic allergenicity using the salt-soluble protein extract (SSPE) from durum, a tetraploid wheat (*Triticum durum*). Here, we validated the model for three other wheat species [hexaploid common wheat (*Triticum aestivum*)*,* diploid einkorn wheat (*Triticum monococcum*), and the ancient diploid wheat progenitor, *Aegilops tauschii*], and then tested the hypothesis that the SSPEs from wheat species will exhibit differences in relative allergenicities. Balb/c mice were repeatedly exposed to SSPEs via the skin. Allergic sensitization potential was assessed by specific (s) IgE antibody responses. Oral anaphylaxis was quantified by the hypothermic shock response (HSR). The mucosal mast cell response (MMCR) was determined by measuring mast cell protease in the blood. While *T. monococcum* elicited the least, but significant, sensitization, others were comparable. Whereas *Ae. taushcii* elicited the least HSR, the other three elicited much higher HSRs. Similarly, while *Ae. tauschii* elicited the least MMCR, the other wheats elicited much higher MMCR as well. In conclusion, this pre-clinical comparative mapping strategy may be used to identify potentially hyper-, hypo- and non-allergenic wheat varieties via crossbreeding and genetic engineering methods.

## 1. Introduction

Food allergies are a critical public health issue at the global level [1]. It currently affects 10.8% of adults and 8% of children in the US [2,3]. Similar trends have been reported by many other countries, such as Canada, the EU, Japan, and Australia [4,5,6,7,8,9]. The annual economic impact of food allergies in the US was estimated to be $24.8 billion in 2013 [10]. Allergic reactions to offending foods can be potentially life-threatening with manifestations of systemic anaphylaxis [11]. Individuals affected by allergies must strictly follow elimination diets, which can reduce their quality of life (e.g., need for hypervigilance; increased risk for anxiety attacks) and cause a significant social burden [3]. 

Wheat has extensive genetic diversity across domesticated and wild species of different ploidy levels. Modern common wheat, *Triticum aestivum* (2n = 6x = 42), is a hexaploid carrying A, B, and D genomes derived from two interspecific hybridization events [12]. Durum wheat, *Triticum durum* (2n = 4x = 28), is a tetraploid carrying A and B genomes. Einkorn wheat, *Triticum monococcum*, is a cultivated diploid A genome species. *Aegilops tauschii* is the D genome donor that hybridized with a tetraploid wheat species to generate modern common wheat [13].

While wheat is a global staple food [14] and a source of essential nutrients including protein, B vitamins, and minerals, consumption can be associated with various adverse health conditions, including: celiac disease, non-celiac gluten/wheat sensitivity, food protein-induced enterocolitis syndrome, eosinophilic esophagitis, wheat food allergy, wheat-dependent exercise-induced anaphylaxis (WDEIA), and contact urticaria. Although all these conditions are caused by the overactivation of the immune system, the last four are IgE antibody-mediated allergic reactions. Approximately 0.2–1.3% of children and 0.9–3.6% of adults in the US have wheat allergies [15,16,17,18,19]. In Europe, wheat allergies affect 1% of children and 0.4% of adults [20,21,22]. Wheat is the third most allergenic food after milk and eggs among the children in Japan [23]. Therefore, wheat is regulated in many countries as a major food allergen along with milk, fish, shellfish, peanuts, tree nuts, eggs, soybean, sesame, celery, lupin, mustard, and sulfites [4,6,7,8,9,24,25,26].

Wheat proteins include gluten and non-gluten fractions. The gluten fraction accounts for 80–85% of the total wheat proteins, whereas 15–20% is the salt-soluble, non-gluten fraction [27]. Both types of proteins have been implicated in causing wheat allergies [28]. Wheat allergy is often confused with celiac disease, an autoimmune disease triggered by gluten proteins [29]. 

The current food allergen regulation per the United States Food and Drug Administration (US FDA) assumes that all wheats, independent of their genetics, are alike in their intrinsic allergenicity potential [26]. The pathogenesis of food allergies involves two distinct sequential phases. IgE production first leads to sensitization during initial exposures, followed by disease elicitation upon subsequent exposures. There are several in vitro studies that suggest that genetically distinct wheats may differ in their IgE binding capacities [30,31,32]. These studies examined the IgE antibody binding to proteins extracted from genetically different wheats using immunoassays. However, whether wheat species differ in their *de novo* sensitization potencies and disease elicitation properties is largely unknown. Thus, in vivo studies are urgently needed to test whether wheat species differ in their intrinsic allergenic properties. Although animal models could be used to address this problem, such studies have not been reported so far.

Several animal models have been reported in the literature to study wheat allergenicity [27,29]. We recently reported a novel adjuvant-free transdermal sensitization followed by oral elicitation (TS/OE) mouse model of wheat allergy using *T. durum* [33]. The TS/OE mouse model is uniquely suited for testing the intrinsic allergenicity potential of genetically distinct wheats because it does not use adjuvants for inducing sensitization to the wheat allergen [33]. Here, we validated the TS/OE model for three other wheat species [hexaploid common wheat (*Triticum aestivum*)*,* diploid einkorn wheat (*Triticum monococcum*), and the ancient diploid wheat progenitor, *Aegilops tauschii*], and then tested the hypothesis that the SSPEs from wheat species will exhibit differences in relative allergenicities. The objectives of this study were to: (i) validate the TS/OE mouse model for allergenic sensitization and oral disease elicitation using SSPE from *T. monococcum*, *T. aestivum*, and *Ae. Tauschii*; and (ii) develop comparative maps of intrinsic allergenicity sensitization and disease elicitation potentials of the four wheat species. Our results demonstrate that this pre-clinical comparative mapping strategy may be used to identify potentially hyper-, hypo- and non-allergenic wheat varieties via crossbreeding and genetic engineering methods.

## 2. Results

### 2.1. Validation of the Transdermal Sensitization and Oral Elicitation of Disease Mouse Model for Triticum monococcum Using Salt-Soluble Protein Extract

#### 2.1.1. Transdermal Exposure to Salt-Soluble Protein Extract from *T. monococcum* Elicits Robust Specific-IgE Antibody Response in Balb/c Mice

**Figure 1:**
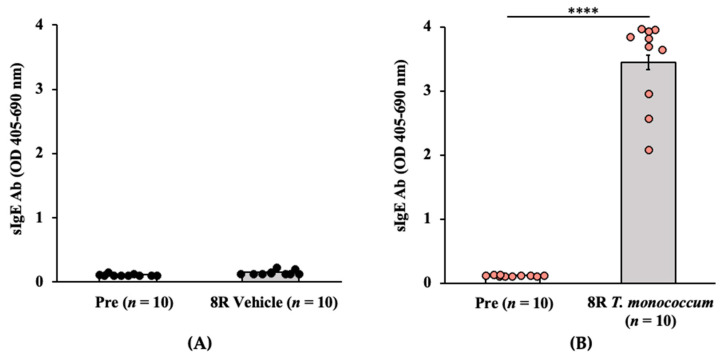
Transdermal exposure of Balb/c mice to SSPE from *Triticum monococcum* (genome AA) elicited robust specific (s) IgE antibody responses. Mice were exposed to *T. monococcum* SSPE or saline as described in Methods. Plasma collected before the 1st exposure (Pre) and after the 8th exposure (8R) was used in the measurement of sIgE levels (OD 405–690 nm). (**A**) sIgE levels in control mice. (**B**) sIgE levels in sensitized mice. **** *p* < 0.001, student’s *t*-test. Ab: antibody; *n*: number of mice; SSPE: salt-soluble protein extract; OD: optical density.

Groups of Balb/c female mice were exposed via the skin to salt-soluble protein extract (SSPE) from diploid *T. monococcum* (Einkorn, genome AA) or to saline by repeated weekly exposures as described in the methods. Blood samples collected before the 1st and after the 8th skin exposures were used in the measurements of specific (s) IgE levels. A robust induction of sIgE antibody levels after transdermal exposure with SSPE but not vehicles was noted (~29-fold increase in allergic mice vs. vehicle control mice) (Figure 1A,B). 

#### 2.1.2. Oral Challenge with *T. monococcum* SSPE Elicits Hypothermic Shock Responses in Skin-Sensitized Mice

**Figure 2:**
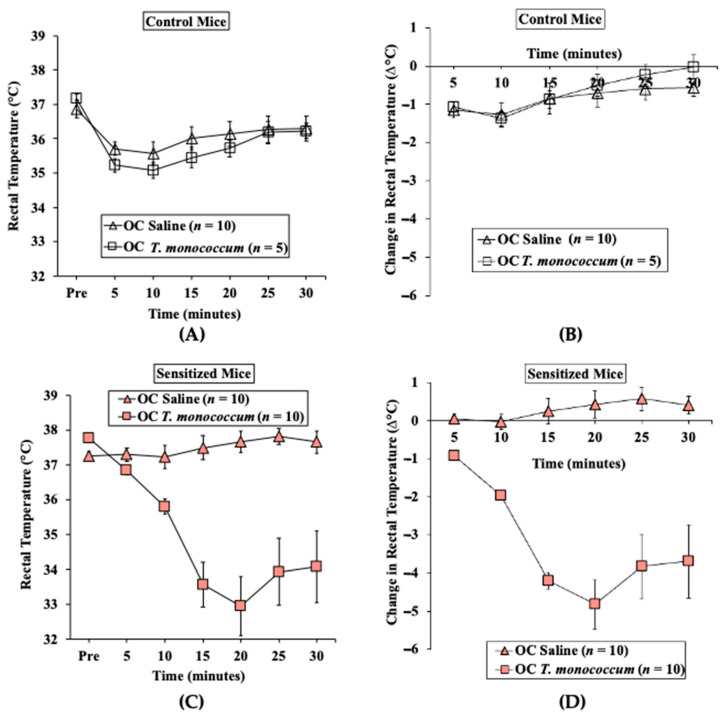
Transdermal sensitization with SSPE is sufficient for eliciting oral anaphylaxis using *Triticum monococcum* (genome AA) SSPE in Balb/c mice. Mice were sensitized and orally challenged with *T. monococcum* SSPE or saline, as described in Materials and Methods. (**A**) Actual rectal temperature at indicated time points in control mice challenged with *T. monococcum* SSPE or saline. (**B**) Change in rectal temperature at indicated time points in control mice challenged with *T. monococcum* SSPE or saline. (**C**) Actual rectal temperature at indicated time points in SSPE-sensitized mice challenged with *T. monococcum* SSPE or saline. (**D**) Change in rectal temperature at indicated time points in SSPE-sensitized mice challenged with *T. monococcum* SSPE or saline. OC: oral challenge, SSPE: salt-soluble protein extract, *n*: number of mice, SSPE: salt-soluble protein extract.

We used parallel groups of skin-sensitized mice to induce anaphylaxis by performing the oral challenge with *T. monococcum* SSPE (20 mg/mouse) or saline. Anaphylactic reactions were quantified by hypothermic shock reactions (HSR) using rectal thermometry, as described in methods. There was no HSR upon vehicle (i.e., zero allergen) or SSPE challenge in control mice (Figure 2A,B). In contrast, acute HSRs were observed upon oral allergen challenge in sensitized mice (Figure 2C,D). Significant HSRs were noted from 15 to 30 min post-oral allergen challenge (ANOVA, *p* < 0.05).

#### 2.1.3. Oral Anaphylaxis Elicited by *T. monococcum* Is Associated with Robust Mucosal Mast Cell Response (MMCR) in Balb/c Mice

**Figure 3:**
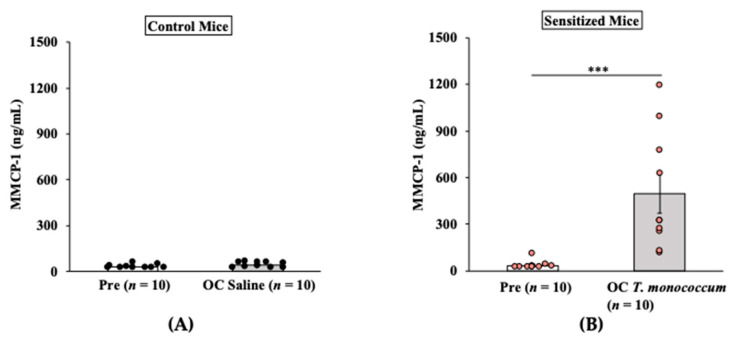
Oral challenge with SSPE from *Triticum monococcum* (genome AA) elicits a robust mucosal mast cell response (MMCR) in Balb/c mice. Mice were sensitized and orally challenged with *T. monococcum* SSPE, or saline as described in Materials and Methods. Plasma levels of mucosal mast cell protease (MMCP)-1 levels (ng/mL) in pre- and 1-h post-challenge were measured by ELISA. (**A**) MMCP-1 levels in control mice challenged with saline. (**B**) MMCP-1 levels in allergic mice challenged with *T. monococcum* SSPE. *** *p* < 0.005, student’s *t*-test. *n*: number of mice, OC: oral challenge, SSPE: salt-soluble protein extract, ELISA: enzyme-linked immunosorbent assay.

It has been shown in a previous study that IgE-mediated systemic anaphylaxis is induced by degranulation of mucosal mast cells, which manifests as an acute elevation of blood levels of murine mucosal cell protease (MMCP)-1 after allergen challenge [34]. Results of MMCP-1 responses in control mice and in allergic (SSPE-sensitized) mice are shown in Figure 3A,B. As evident, oral challenge with *T. monococcum* SSPE (20 mg/mouse) but not saline induced a marked elevation of MMCP-1 levels in the blood (Figure 3A,B).

### 2.2. Validation of Transdermal Sensitization and Oral Elicitation of Disease Model Using Salt-Soluble Protein Extract from Triticum aestivum

#### 2.2.1. Transdermal Exposure to Salt-Soluble Protein Extract from *T. aestivum* Elicits Robust Specific-IgE Antibody Response in Balb/c Mice

**Figure 4:**
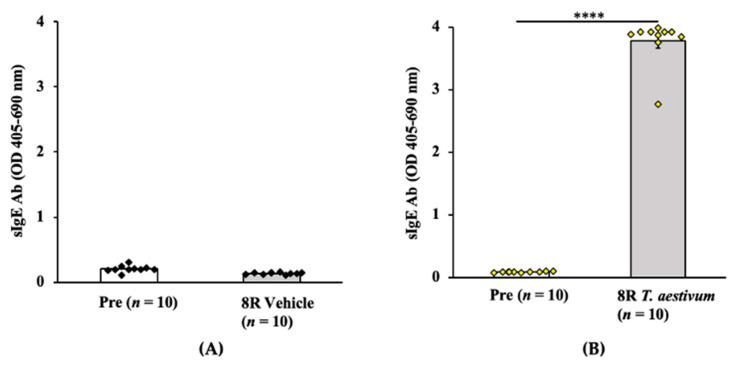
Transdermal exposure of Balb/c mice to SSPE from *Triticum aestivum* (genomes AABBDD) elicited robust specific (s) IgE antibody responses. Mice were exposed to *T. aestivum* SSPE, or saline as described in Methods. Plasma collected before the 1st exposure (Pre) and after the 8th exposure (8R) was used in the measurement of sIgE levels (OD 405–690 nm). (**A**) sIgE levels in control mice. (**B**) sIgE levels in sensitized mice. **** *p* < 0.001, student’s *t*-test. Ab: antibody, *n*: number of mice, SSPE: salt-soluble protein extract.

Groups of Balb/c female mice were exposed via the skin to salt-soluble protein extract (SSPE) from *T. aestivum* (genomes AABBDD) [35] or saline by repeated weekly exposures as described in the methods. Blood collected before the 1st and after the 8th skin exposure was used in the measurements of specific (s) IgE levels. As can be seen in Figure 4A,B, a robust induction of sIgE antibody levels after transdermal exposure with *T. aestivum* SSPE but not vehicle exposure was noted (~43-fold increase in sensitized mice vs. vehicle control mice).

#### 2.2.2. Oral Challenge with *T. aestivum* SSPE Elicits Hypothermic Shock Responses in Skin-Sensitized Mice

**Figure 5:**
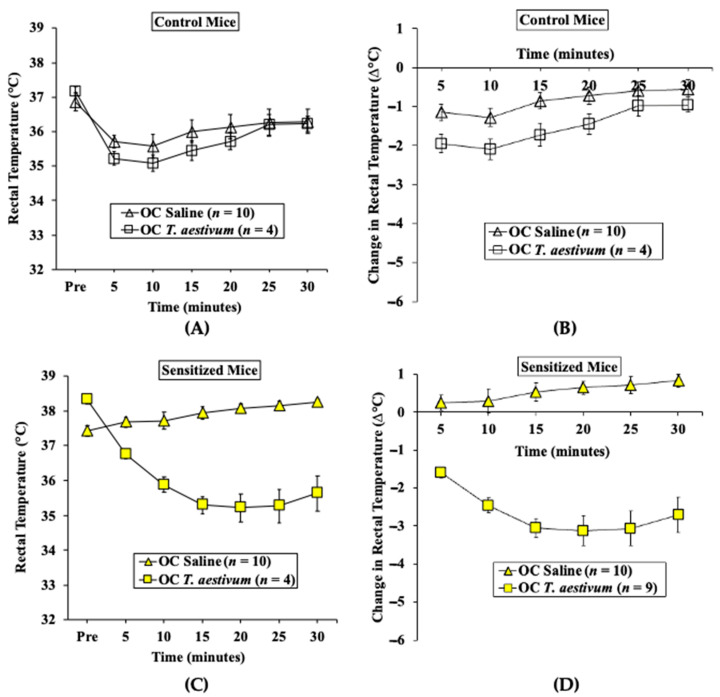
Transdermal sensitization with SSPE is sufficient for eliciting oral anaphylaxis using *Triticum aestivum* SSPE in Balb/c mice. Mice were sensitized and orally challenged with *T. aestivum* SSPE or with saline, as described in Materials and Methods. (**A**) Actual rectal temperature at indicated time points in control mice challenged with *T. aestivum* SSPE or saline. (**B**) Change in rectal temperature at indicated time points in control mice challenged with *T. aestivum* SSPE or saline. (**C**) Actual rectal temperature at indicated time points in control mice challenged with *T. aestivum* SSPE or saline. (**D**) Change in rectal temperature at indicated time points in allergic mice challenged with *T. aestivum* SSPE or saline. Ab: antibody, OC: oral challenge, SSPE: salt-soluble protein extract, *n*: number of mice, OC: oral challenge, SSPE: salt-soluble protein extract.

We used parallel groups of skin-sensitized mice to induce anaphylaxis by performing an oral challenge with SSPE (20 mg/mouse) or saline. Anaphylactic reactions were quantified by hypothermic shock reactions (HSRs) using rectal thermometry as described in the methods. There was no HSR upon vehicle (i.e., zero allergen) or SSPE challenge in control mice (Figure 5A,B). In contrast, acute HSRs were observed upon oral allergen challenge in sensitized mice (Figure 5C,D). Significant HSRs were noted from 15 to 30 min post-oral allergen challenge (ANOVA, *p* < 0.05).

#### 2.2.3. Oral Anaphylaxis Elicited by *T. aestivum* SSPE Is Associated with Robust Mucosal Mast Cell Response (MMCR) in Balb/c Mice

**Figure 6:**
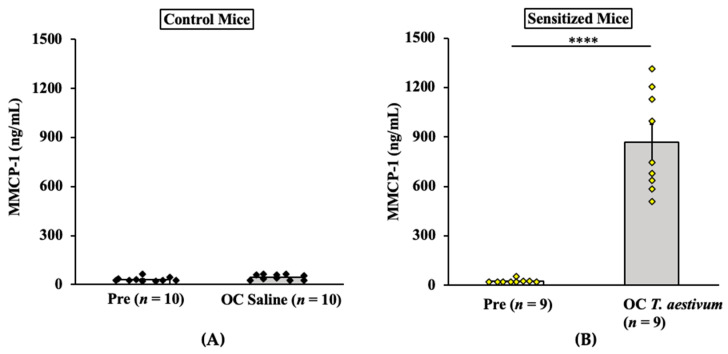
Oral challenge with SSPE from *Triticum aestivum* elicits a robust mucosal mast cell response (MMCR) in Balb/c mice. Mice were sensitized and orally challenged with *T. aestivum* SSPE or saline, as described in Materials and Methods. Plasma levels of mucosal mast cell protease (MMCP)-1 levels (ng/mL) in pre- and 1-h post-challenge were measured by ELISA. (**A**) MMCP-1 levels in control mice challenged with saline. (**B**) MMCP-1 levels in allergic mice challenged with *T. aestivum* SSPE. **** *p* < 0.005, student’s *t*-test. *n*: number of mice, OC: oral challenge, SSPE: salt-soluble protein extract, ELISA: enzyme-linked immunosorbent assay.

Results of MMCP-1 responses in control mice and allergic mice are shown in Figure 6A,B. As evident, oral challenge with *T. aestivum* SSPE (20 mg/mouse) but not saline induced a marked elevation of MMCP-1 levels in the blood (Figure 6A,B).

### 2.3. Validation of Transdermal Sensitization and Oral Elicitation of Disease Model Using Salt-Soluble Protein Extract from Aegilops tauschii

#### 2.3.1. Transdermal Exposure to Salt-Soluble Protein Extract from *Aegilops tauschii* Elicits Robust Specific-IgE Antibody Response in Balb/c Mice

**Figure 7:**
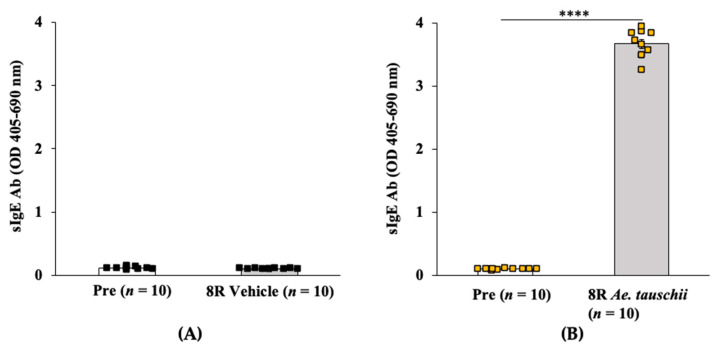
Transdermal exposure of Balb/c mice to SSPE from *Aegilops tauschii* elicited robust specific (s) IgE antibody responses. Mice were exposed to *Ae. tauschii* SSPE or saline as described in Methods. Plasma collected before the 1st exposure (Pre) and after the 8th exposure (8R) was used in the measurement of sIgE levels (OD 405–690 nm). (**A**) sIgE levels in control mice. (**B**) sIgE levels in sensitized mice. **** *p* < 0.001, student’s *t*-test. Ab: antibody, *n*: number of mice, SSPE: salt-soluble protein extract.

Groups of Balb/c female mice were exposed via the skin to salt-soluble protein extract (SSPE) from the hexaploid *Ae. tauschii* (genome DD) or saline by repeated weekly exposures as described in the methods. Blood collected before the 1st and after the 8th skin exposures was used in the measurements of specific (s) IgE levels. As can be seen in Figure 7A,B, a robust induction of sIgE antibody levels after transdermal exposure with SSPE but not vehicles was noted (~36-fold increase in sensitized mice vs. vehicle control mice).

#### 2.3.2. Oral Challenge with *Ae. tauschii* SSPE Elicits Hypothermia Shock Responses in Skin-Sensitized Mice

**Figure 8:**
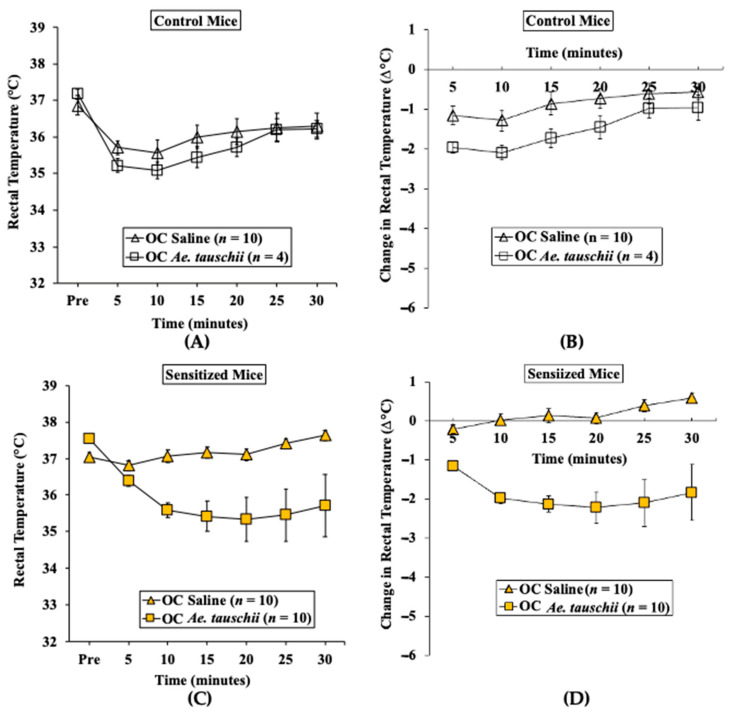
Transdermal sensitization with SSPE is sufficient for eliciting oral anaphylaxis using *Aegilops tauschii* SSPE in Balb/c mice. Mice were sensitized and orally challenged with *Ae. tauschii* SSPE or with saline as described in Materials and Methods. (**A**) Actual rectal temperature at indicated time points in control mice challenged with *Ae. tauschii* SSPE or saline. (**B**) Change in rectal temperature at indicated time points in control mice challenged with *Ae. tauschii* SSPE or saline. (**C**) Actual rectal temperatures at indicated time points in SSPE-sensitized mice challenged with *Ae. tauschii* SSPE or saline. (**D**) Change in rectal temperature at indicated time points in SSPE-sensitized mice challenged with *Ae. tauschii* SSPE or saline. Ab: antibody, OC: oral challenge, SSPE: salt-soluble protein extract, *n*: number of mice, OC: oral challenge, SSPE: salt-soluble protein extract.

We used parallel groups of skin-sensitized mice to induce anaphylaxis by performing the oral challenge with *Ae. tauschii* SSPE (20 mg/mouse) or saline. Anaphylactic reactions were quantified by hypothermic shock reactions (HSRs) using rectal thermometry as described in the methods. There was no HSR upon vehicle (i.e., zero allergen) or SSPE challenge in control mice (Figure 8A,B). In contrast, acute HSRs were observed upon oral SSPE allergen challenge in sensitized mice (Figure 8C,D). Significant HSRs were noted from 15 to 30 min post-oral allergen challenge (ANOVA, *p* < 0.05).

#### 2.3.3. Oral Anaphylaxis Elicited by *Ae. tauschii* Is Associated with Robust Mucosal Mast Cell Response (MMCR) in Balb/c Mice

**Figure 9:**
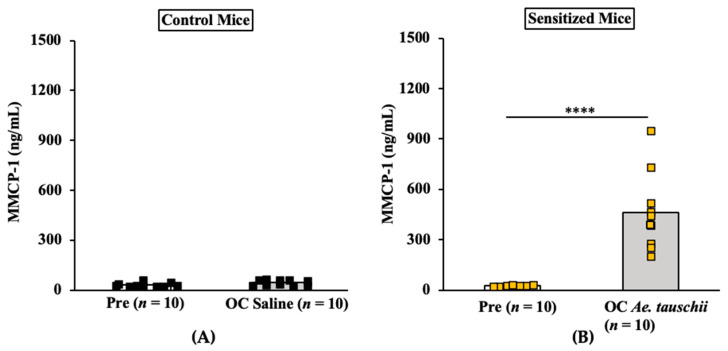
Oral challenge with SSPE from *Aegilops tauschii* elicits a robust mucosal mast cell response (MMCR) in Balb/c mice. Mice were sensitized and orally challenged with *Ae. tauschii* SSPE or saline as described in the Materials and Methods. Plasma levels of mucosal mast cell protease (MMCP)-1 (ng/mL) in pre- and 1-h post-challenge were measured by ELISA. (**A**) MMCP-1 levels in control mice challenged with saline. (**B**) MMCP-1 levels in allergic mice challenged with *Ae. tauschii* SSPE. **** *p* < 0.005, student’s *t*-test, *n*: number of mice, OC: oral challenge, SSPE: salt-soluble protein extract, ELISA: enzyme linked immunosorbent assay.

Results of MMCP-1 responses in control mice and allergic mice are shown in Figure 9A,B. As evident, oral challenge with *Ae. tauschii* SSPE (20 mg/mouse), but not saline, induces marked elevation of MMCP-1 levels in the blood (Figure 9A,B).

### 2.4. Comparative Map of the Intrinsic Allergenicity Sensitization Potential of Salt-Soluble Protein Extracts from the Diploid, Tetraploid, and Hexaploid Wheat

**Figure 10:**
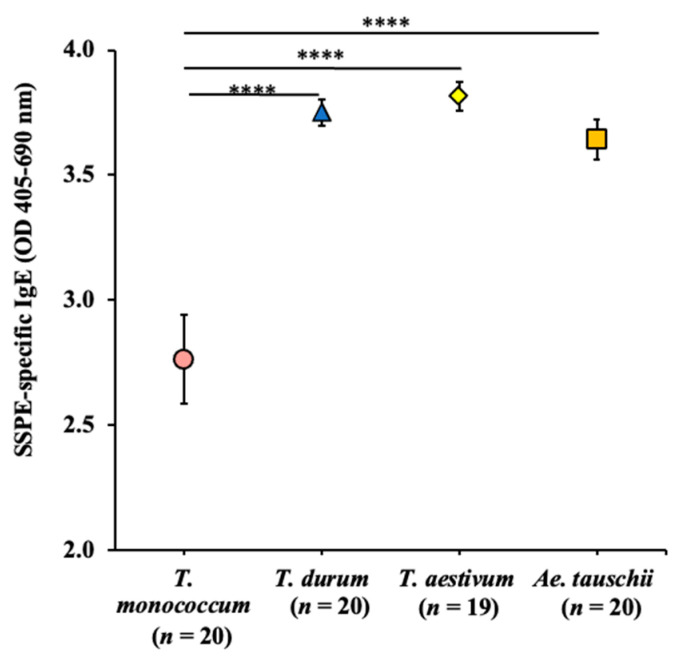
A comparative map of the intrinsic allergenicity sensitization potential of SSPEs from diploid, tetraploid, and hexaploid wheats. The changes in SSPE-specific IgE antibody (Ab) levels after the 8th skin exposure to SSPEs from the respective wheats are shown in the figure. **** *p* < 0.001, one-way ANOVA and Tukey’s post hoc tests. *n*: number of mice, SSPE: salt-soluble protein extract.

We used the sIgE data from the above validation studies and our previously reported durum wheat study [33] for preparing a comparative sensitization map. The sIgE antibody levels elicited by respective wheats were determined by subtracting the baseline (pre) sIgE levels from the 8th response (8R) sIgE levels. The resulting comparative map of the intrinsic allergenicity and sensitization potential of the four genetically distinct wheats is shown in Figure 10. *T. durum* and *T. aestivum* SSPEs elicited almost identical sIgE levels, and *Ae. tauschii* elicited slightly lower sIgE levels. *T. monococcum* (genome AA) elicited significantly lower sIgE levels than the other three wheats.

### 2.5. Comparative Map of the Intrinsic Allergenicity Disease Elicitation Potential of Salt-Soluble Protein Extracts from the Diploid, Tetraploid, and Hexaploid Wheats

**Figure 11:**
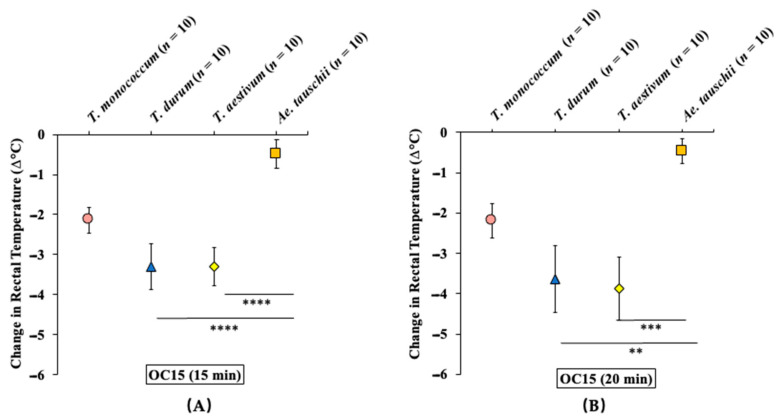

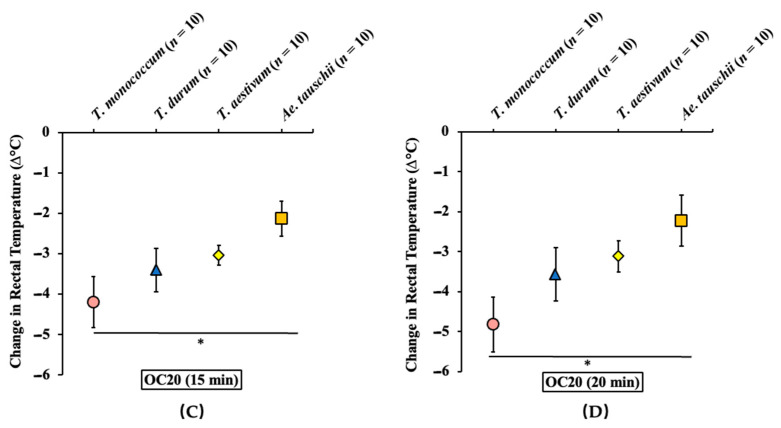
Comparative map of the intrinsic allergenicity disease elicitation potential of SSPEs from diploid, tetraploid, and hexaploid wheat. (**A**,**B**) HSRs at 15 min and 20 min after oral challenge doses of 15 mg (OC15) SSPE of diploid, tetraploid, and hexaploid wheat. (**C**,**D**) HSRs at 15 min and 20 min after oral challenge doses of 20 mg (OC20) of diploid, tetraploid, and hexaploid wheat. * *p* < 0.05, ** *p* < 0.01, *** *p* < 0.005, **** *p* < 0.001, one-way ANOVA and Tukey’s post hoc tests. *n*: number of mice, SSPE: salt-soluble protein extract, HSR: hypothermic shock response.

We used the absolute changes in the rectal temperature data upon oral allergen challenge from the above validation studies of *T. monococcum*, *T. aestivum* and *Ae. tauschii* wheat and our previously reported durum wheat (*T. durum*) studies [33] for preparing a comparative disease elicitation map. Figure 11A,B show the disease elicitation potential map at 15 and 20 min post oral allergen challenge with a 15 mg SSPE dose. Figure 11C,D show the disease elicitation potential map at 15 and 20 min post-oral allergen challenge with a 20 mg dose. As is evident, *Ae. tauschii* elicited the least HSR compared to the other wheat species. *T. monococcum* elicited lower HSR responses compared to *T. durum* and *T. aestivum* at a 15 mg dose but not at a 20 mg dose (Figure 11A–D). Similar disease elicitation potential maps were obtained for 25 and 30 min post-oral allergen challenge time points (data not shown).

### 2.6. Comparative Map of the Mucosal Mast Cell Response (MMCR) Elicitation Potential of Salt-Soluble Protein Extracts from the Diploid, Tetraploid, and Hexaploid Wheats

**Figure 12:**
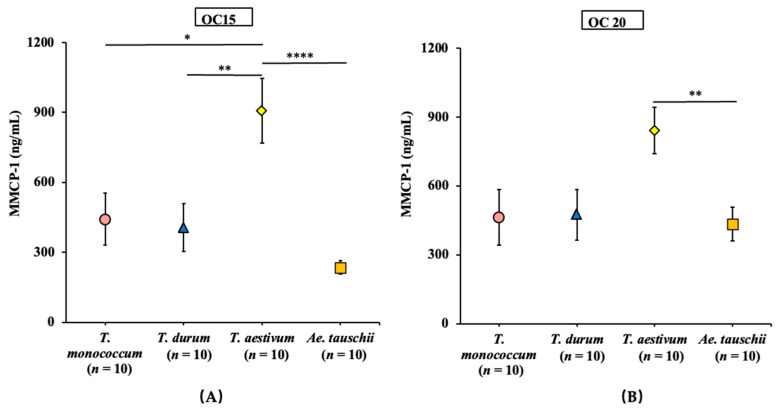
Comparative map of the mucosal mast cell response (MMCR) elicitation potential of SSPEs from diploid, tetraploid, and hexaploid wheats. (**A**) Average MMCP-1 blood level after 15 mg oral SSPE allergen challenge dose (OC15). (**B**) Average MMCP-1 blood level after a 20 mg oral allergen challenge dose (OC20). * *p* < 0.05, ** *p* < 0.01, **** *p* < 0.001, one-way ANOVA and Tukey’s post hoc tests. OC: oral challenge. MMCP-1: mucosal mast cell protease-1. *n*: number of mice, OC: oral challenge, SSPE: salt-soluble protein extract, MMCP-1: mucosal mast cell protease-1.

We used the MMCP-1 data upon oral allergen challenge from the above validation studies of *T. monococcum*, *T. aestivum*, *Ae. tauschii* wheats and our previously reported durum wheat (*T. durum*) studies (Gao et al., 2022) for preparing a comparative MMCR elicitation potential map. Figure 12A,B show the MMCR elicitation potential maps at 15 and 20 mg oral allergen challenge doses, respectively. As evident, *T. aestivum* elicited the highest MMCR, followed by *T. monococcum* and *T. durum,* which were comparable to each other. *Ae. tauschii* elicited the lowest MMCR, which was significantly lower than that of *T. aestivum* but not of *T. monococcum* or *T. durum*.

## 3. Discussion

Here, we validated the TS/OE mouse using salt-soluble protein extracts (SSPEs) from einkorn wheat, common wheat and the ancient wheat progenitor tauschii, and then tested the hypothesis that the SSPEs from four wheat species (hexaploid *T. aestivum*, tetraploid *T. durum*, diploid *T. monococcum* and *Ae. tauschii* wheat) will exhibit differences in intrinsic allergenicity potential. We were uniquely positioned to test this hypothesis because we recently published a novel adjuvant-free transdermal sensitization/oral elicitation (TS/OE) mouse model using durum wheat that could be used as a pre-clinical tool to address this hypothesis [33]. We report the first comparative map illustrating the intrinsic allergenicity potential of these wheat species of differing ploidy levels.

This research reports four novel findings: (i) validation of the TS/OE mouse model for allergenic sensitization and oral disease elicitation using SSPEs from four wheat species; (ii) development of a comparative map of the intrinsic allergenicity sensitization potential of *T. aestivum*, *T. durum*, *T. monococcum*, and *Ae. tauschii*; (iii) development of a comparative map of the intrinsic hypothermia shock response (HSR) (i.e., systemic anaphylaxis) elicitation potential of these four wheat species; and iv) development of a comparative map of mucosal mast cell response (MMCR) elicitation potential of these four wheats. Thus, this work has not only validated the TS/OE mouse model for genetically distinct hexaploid and diploid wheats but also provided pre-clinical intrinsic allergenicity potential maps of diploid, tetraploid, and hexaploid wheats.

We chose wheat species representing the wheat diploid AA, DD, tetraploid AABB and hexaploid AABBDD genomes. We previously developed and characterized the TS/OE mouse model using SSPE from the durum wheat variety Carpio [36]. The *T. aestivum* wheat variety Ambassador used in this study is a hexaploid common wheat (genomes AABBDD) that is mostly used for cracker and cookie making [37]. *T. monococcum* is a cultivated form of the A genome species, known as einkorn wheat; it is commercially available, and there is significant research interest in characterizing its health promoting properties [38]. *Ae. tauschii* is the D genome donor to modern common wheat, and no cultivated commercial forms are currently available. It is noteworthy that although we chose one representative variety from each genotype in this study, future research is needed to verify whether or not the chosen variety might represent most other varieties within each wheat species for intrinsic allergenicity potential [39].

Compared to other animal models (i.e., dog, rat, and swine), mouse models have several advantages, including relatively low cost of purchase and management and ready availability of reagents [27]. Most previously published mouse models of wheat allergy are adjuvant-based, which reflect a situation of co-exposure to both allergens and adjuvants, and they are very useful to study mechanisms of disease. However, they are not considered suitable to evaluate the intrinsic allergenicity of wheat proteins because the adjuvants are thought to enhance sensitivity and reduce specificity [27,40]. For example, the intrinsic allergenicity property of wheat proteins independent of the effect of an adjuvant is difficult to decipher from such models. Therefore, adjuvant-based models do not have the capability to reveal the intrinsic allergenicity of wheat proteins. On the contrary, an adjuvant-free mouse model is preferred to address this issue, as it will make the data interpretation much easier without the need to differentiate the effect of the adjuvant from that of allergens. Gangur and coworkers have developed a novel adjuvant-free transdermal sensitization and oral elicitation (TS/OE) mouse model of food allergy that is capable of simulating many aspects of human food allergies [40]. This model has been utilized in assessing the intrinsic allergenicity of multiple food allergens (e.g., shellfish, tree nuts, eggs, milk, and sesame), and recently wheat [33,41]. Therefore, we employed the TS/OE mouse model in this study.

In this study, we tested sIgE levels before exposure vs. after the eigth exposure to the SSPEs. We also analyzed the sIgE after the sixth exposure and found that there was a progressive and continuous increment in sIgE levels. There are two previous studies that demonstrated the potential variations in in vitro sIgE-binding allergenicity among different wheat lines/varieties. In one study, researchers tested the IgE binding capacity of several wheat varieties, including diploid (*T. monococcum*), tetraploid (*T. durum, T. dicoccum, T. polonicum, T. turgidum*) and hexaploid (*T. aestivum, T. compactum, T. spelta*) wheats [30]. They used direct IgE ELISA to characterize the allergenicity of 324 wheats, among which several candidates, including Einkorn (*T. monococcum*), were identified as less allergenic based on binding to IgE antibodies obtained from wheat-allergic patients. They found that the IgE reactivities of tetraploid and hexaploid wheats were higher than those of diploid Einkorn wheat. These data concur in principle with our findings that *T. monococcum* (Einkorn) elicited lower IgE production responses compared to the tetraploid durum wheat in our TS/OE mouse model. In another study, researchers compared the IgE-binding capacity of salt-soluble protein extracts (SSPE) from the hexaploid wheat (*T. aestivum*, cultivar Récital) with that of the diploid wheat Engrain (*T. monococcum*, genome AA) [32]. Although they used different varieties of hexaploid and diploid wheats than that we have used in this mouse model study, results are consistent in that diploid A genome wheat species demonstrate lower IgE binding capacity than the hexaploid wheat.

The grains of the four tested species were obtained in different ways. That is, the environments in which they were grown were different. For example, durum wheat (variety Carpio) was grown in North Dakota, soft wheat (Ambassador) was grown in Michigan, the ancient *Ae. tauschii* wheat was grown in a greenhouse at Michigan State University, and the Einkorn wheat was purchased from a commercial source (einkorn.com). The growing environment can affect the quantity of wheat proteins in each species. The four wheat species evaluated in this study carry unique genomes and produce grains containing unique proteins. Different growing environments mainly affect the protein content (i.e., quantity), but not the types of proteins present. We characterized the protein extracts for their quality by SDS-PAGE analysis. We used an identical quantity of each protein extract in our experiments. Since our tests were focused on the types of proteins present in the four flour samples (for their allergenicities) rather than the quantity of the proteins in the flour samples, the different growing environments among the four wheat lines should have a minimal effect, if any, on the allergenicities of the tested wheat lines. There is some evidence in the literature that reports differences in the types of salt-soluble allergenic proteins in the diploid wheat Engrain (*T. monococcum*, genome AA) vs. the hexaploid wheat *T. aestivum* (cultivar Récital, genome AABBDD) [32]. They demonstrated the absence of certain allergens in the former.

We noted that *T. monococcum* elicited significantly lower sIgE levels. Similar findings were reported in human wheat-allergic subjects who also produced lower sIgE levels for the diploid wheat Engrain, *T. monococcum (genome AA)* compared to the hexaploid wheat *T. aestivum* (cultivar Récital, genome AABBDD) [32]. We used an identical quantity of the protein as the other wheat proteins. There is some evidence that *T. monoccum* does not contain certain types of allergens that are present in the hexaploid wheat *T. aestivum* (cultivar Récital) [32]. Therefore, it is possible that the diploid wheat einkorn (*T. monococcum*, genome AA) used in this study might lack one or more allergenic proteins that are present in other wheats. Future studies may consider testing this hypothesis.

We noted that *T. aestivum* elicited the highest mucosal mast cell response after the oral allergen challenge. We used an identical quantity of the protein as the other wheat proteins for the oral challenges. We speculate two possibilities: (i) the SSPE from *T. aestivum* might survive the gastric/enteric digestion more than the other wheat genotypes and therefore cause a stronger mucosal mast cell response after oral allergen challenge; (ii) the SSPE from *T. aestivum* may contain adjuvant-like components that enhance the mucosal mast cell response to oral allergen challenge. Future studies may consider testing these hypotheses.

As discussed above, the four wheat species evaluated in this study carry unique genomes and produce grains containing unique proteins. Our goal was to test if differences in genotypes result in differences in the allergenicity of their proteins. Specific differences in the types of protein allergens present in the four genotypes might explain the differences in elicited allergenicity in this study. We also noted that for *T. monococcum,* the higher dose used in oral challenge impacted a big change in the hypothermic shock response. We do not know the underlying reason, but we speculate that this might be related to the potency characteristics of the specific allergens present in that wheat.

When fractionated, wheat proteins are composed of salt-insoluble glutens and salt-soluble non-gluten proteins. Both protein fractions act as allergens and trigger wheat allergy symptoms in humans [28]. In this study, we focused on investigating the allergenicity of non-gluten SSPEs, as they are understudied compared to the gluten proteins. In addition, none of the studies in the past have compared the in vivo allergenicity potential of SSPEs from four genetically different wheat species. Therefore, here we validated the TS/OE model and developed intrinsic allergenicity potential maps using SSPEs from four distinct wheat species. A similar approach could be used to develop intrinsic allergenicity maps for glutens from diploid, tetraploid, and hexaploid wheats.

Many studies have shown that food processing can alter the allergenicity of food proteins, including those of wheat [29,42,43]. For instance, wheat allergens under novel processing methods may have new epitopes generated or hidden epitopes revealed, either of which may increase their allergenicity. The intrinsic allergenicity maps could be developed using processed wheats by applying the approach presented here. By comparing the allergenicity potential maps of native vs. processed wheat proteins, it is possible to determine the quantitative effects of processing on intrinsic allergenicity within each genotype of wheat. Such work has the potential to identify and tailor specific processing conditions to produce hypo- and non-allergenic wheat products within a particular genetic background. At the same time, using comparative potential maps of allergenicity, it is also possible to identify and remove potentially hyper-allergenic wheats and protect wheat-sensitive consumers.

In summary, we report the first utilization of an adjuvant-free mouse model as a pre-clinical testing tool for assessing the natural variation in the intrinsic allergenic potential among diploid, tetraploid, and hexaploid wheats. We demonstrate for the first-time relative differences in the intrinsic allergenicity potential among 4 wheat species of different ploidy levels. This pre-clinical comparative mapping strategy may be used to identify potentially hyper-, hypo- and non-allergenic wheat varieties via crossbreeding and genetic engineering.

## 4. Materials and Methods

### 4.1. Chemicals and Reagents

Biotin-conjugated rat anti-mouse IgE-paired antibodies were obtained from BD BioSciences (San Jose, CA, USA). Streptavidin alkaline phosphatase was obtained from Jackson ImmunoResearch (West Grove, PA, USA). BSA standard (at 2 mg/mL) was purchased from Sigma (St. Louis, MO, USA). p-nitro-phenyl phosphate was obtained from Sigma (St. Louis, MO, USA). Alkaline copper tartrate was purchased from BioRad (Hercules, CA, USA). Folin reagent was purchased from BioRad (Hercules, CA, USA). The following reagents were obtained from Invitrogen (Waltham, MA, USA): IgE Mouse Uncoated ELISA Kit with Plates; Streptavidin-HRP, TMB substrate; MCPT-1 (mMCP-1) Mouse Uncoated ELISA Kit with Plates; Avidin-HRP, TMB substrate. Tissue Protein Extraction Reagent [T-PERTM, a proprietary detergent in 25 mM bicine and 150 mM sodium chloride (pH 7.6)] was from ThermoFisher Scientific (Waltham, MA, USA). A protease (serine, cysteine, acid proteases and aminopeptidases) inhibitor cocktail was obtained from Sigma-Aldrich (St. Louis, MO, USA).

### 4.2. Generation of a Plant-Protein-Free Mouse Colony

Adult Balb/c breeding pairs were purchased from The Jackson Laboratory (Bar Harbor, ME, USA) and were acclimated for a week upon arrival. Each male was paired with 2 females. Female pups aged 6–8 weeks were used in the experiments. All mice were maintained on a plant-protein-free diet (AIN-93G, Envigo, Indianapolis, IN, USA) throughout the study. Animal procedures were in accordance with Michigan State University policies.

### 4.3. Preparation of Salt-Soluble Protein Extract from Wheat Flours

The following wheats were used in the study: Einkorn (*Triticum monococcum*, genome AA, 2n = 2x = 14), durum (*Triticum durum*, cv. Carpio, genomes AABB, 2n = 4x = 28), common wheat (*Triticum aestivum*, cv. Ambassador, genomes AABBDD, 2n = 6x = 42), and *Aegilops tauschii* (*Ae. tauschii*, genome DD) wheats. *Ae. tauschii* was grown at Michigan State University greenhouses with the help of Dr. Eric Olson. Common wheat and durum wheat were obtained from the MSU Wheat Breeding Program and North Dakota State University, respectively. Einkorn wheat was purchased from a commercial source (einkorn.com).

Durum and common wheat flours were prepared using standard methods established in the cereal sciences laboratory (benchtop mill Quadrumat Junior, Brabender, Germany, and roller mill Buhler MLU-202 Mill, Buhler, Switzerland). Einkorn flour was prepared using the equipment and instructions provided by the grain supplier (einkorn.com, benchtop mill Mockmill 100, Wolfgang Mock GmbH, Germany). The spikelets of *A. tauschii* grown at our greenhouse were collected, and the husks were manually removed using forceps to collect the grains. The flour was prepared using a grinder (80335R, Hamilton Beach, Glen Allen, VA, USA), as the quantity of this ancient wheat progenitor material was very limited.

Salt-soluble protein extracts (SSPE) were prepared from the flours of the four wheats above using a published method [36]. Briefly, wheat flours were mixed with 0.5 M NaCl in a 1:10 ratio (*m*/*v*) and stirred continuously for 2 h followed by centrifugation (5000× *g*, 10 min) at 20 °C. The supernatant was collected and stored at −70 °C overnight, followed by freeze-drying the next day. Lyophilized SSPE powder was mixed with sterile saline prior to use for skin sensitization. The protein concentration of the mixture was determined using the Bio-Rad method and was adjusted to 10 mg/mL [37].

### 4.4. Skin Sensitization, Bleeding, and Plasma Sample Preparation

Adult female mice (4–10 per group as specified in the respective experiments) were used in the experiments. Their rump hair was removed using a hair clipper (Philips, Amsterdam, Netherlands). For each mouse, fifty microliters of durum wheat SSPE (10 mg/mL) or vehicle (10% sterile NaCl solution) was applied over both sides of the clipped area (1 mg/100 µL/mouse). Mice were then covered with a non-latex bandage (Johnson & Johnson, New Brunswick, NJ, USA) for one day. The same procedure above was repeated once a week for nine weeks. Bleeding was done through the saphenous vein one week before the 1st exposure (Pre) and after the 8th exposure (8R). Blood was collected into anti-coagulant (lithium heparin)-coated tubes (Sarstedt Inc. MicrovetteCB 300 LH, Numbrecht, Germany) and centrifuged to harvest the plasma. Individual plasma samples were stored at −70 °C until used in analysis.

### 4.5. Elicitation of Oral Anaphylaxis and Hypothermic Shock Responses

Two weeks after the 8th exposure to saline or to wheat SSPE, mice were orally gavaged with vehicle (300 µL sterile saline) or with 15 mg or 20 mg wheat SSPE by using curved feeding needles (22-gauge, length: 1.4 inch, Kent Scientific, Torrington, CT, USA). Mice were monitored for rectal temperature before challenge (Pre) and every 5 min up to 30 min after challenge by using a thermometer with a probe (DIGI-SENSE, Vernon Hills, IL, USA). Actual temperatures (°C) and change in rectal temperature (∆°C) at every 5 min compared to the pre-temperatures for each mouse were used in analyses.

### 4.6. Measurement of Wheat SSPE-Specific IgE Antibody Levels

Wheat SSPE-specific(s) IgE antibody levels in blood samples were measured using an ELISA-based method we previously reported [38,39,40]. This method was a modified version of the published method we have previously reported for food-specific IgE antibody measurement in the mouse system [41]. Briefly, 96-well plates (Corning 3369) were coated with wheat SSPE, followed by blocking (5% gelatin), washing, plasma addition, washing, addition of biotin-conjugated anti-mouse IgE antibody, washing, and addition of Streptavidin Alkaline Phosphatase and PNPP for colorimetry as described before [38,39]. Tests were done in quadruplicate for samples from each mouse.

### 4.7. Quantification of Mucosal Mast Cell Protease-1 (MMCP-1) Level

MMCP-1 levels (ng/mL) in the plasma at 1-h post-challenge were determined using an ELISA-based method per Invitrogen as described previously [39,40]. Briefly, 96-well plates (Corning Costar 9018) were coated with capture antibody (anti-mouse MMCP-1), followed by the addition of samples and standards (recombinant mouse MMCP-1). A sandwich was then formed when a secondary antibody (biotin-conjugated anti-mouse MMCP-1) was added. The detection was based on the avidin-HRP and TMB substrate systems. Assay sensitivity: 120 pg/mL. The standard range used for quantification: 120 to 15,000 pg/mL. Tests were done in quadruplicate for samples from each mouse.

### 4.8. Statistics

An online software service was used in these analyses (https://www.socscistatistics.com/tests/ accessed on multiple days during 1 January 2021 to 30 December 2022). The statistical significance level was set at 0.05. A student’s *t*-test was used to compare two groups, and a one-way ANOVA with post-hoc Tukey HSD was used for multiple comparisons.

## 5. Conclusions

We demonstrate for the first time similarities and differences in the intrinsic allergenicity among the four selected genetically distinct wheat species. This pre-clinical comparative mapping strategy may be used to identify potentially hyper-, hypo- and non-allergenic wheat varieties via crossbreeding and genetic engineering. It can also be used to assess changes in the allergenicity of wheat proteins caused by processing methods.

## Data Availability

All the data are included in the paper. Additional data will be provided as deemed appropriate by the university’s policies.

## References

[1] Sampson H.A., O’Mahony L., Burks A.W., Plaut M., Lack G., Akdis C.A. (2018). Mechanisms of food allergy. J. Allergy Clin. Immunol..

[2] Gupta R.S., Warren C.M., Smith B.M., Blumenstock J.A., Jiang J., Davis M.M., Nadeau K.C. (2018). The Public Health Impact of Parent-Reported Childhood Food Allergies in the United States. Pediatrics.

[3] Gupta R.S., Warren C.M., Smith B.M., Jiang J., Blumenstock J.A., Davis M.M., Schleimer R.P., Nadeau K.C. (2019). Prevalence and Severity of Food Allergies Among US Adults. JAMA Netw. Open.

[4] European Food Safety Authority (2014). Scientific Opinion on the evaluation of allergenic foods and food ingredients for labelling purposes. EFSA J..

[5] U.S. Food & Drug Administration (2022). Food Allergies. https://www.fda.gov/food/food-labeling-nutrition/food-allergies.

[6] Food Standards Australia New Zealand (2021). Food Allergies and Food Intolerances. https://www.foodstandards.gov.au/consumer/foodallergies/allergies/Pages/default.aspx.

[7] Health Canada (2021). Food Allergen Labeling. https://www.canada.ca/en/health-canada/services/food-nutrition/food-labelling/allergen-labelling.html.

[8] (2019). Japan. Food Allergy Labeling. https://expatsguide.jp/articles/features/food-allergy-labeling/.

[9] UK Food Standards Agency (2021). Food Allergy and Intolerance. https://www.food.gov.uk/safety-hygiene/food-allergy-and-intolerance.

[10] Gupta R., Holdford D., Bilaver L., Dyer A., Holl J.L., Meltzer D. (2013). The Economic Impact of Childhood Food Allergy in the United States. JAMA Pediatr..

[11] Warren C.M., Jiang J., Gupta R.S. (2020). Epidemiology and Burden of Food Allergy. Curr. Allergy Asthma Rep..

[12] Sears E.R. (1952). Homoeologous chromosomes in Triticum aestivum. Genet.

[13] McFadden E.S., Sears E.R. (1946). The Origin of Triticum Spelta and Its Free-Threshing Hexaploid Relatives. J. Hered..

[14] Shewry P.R., Hey S.J. (2015). The contribution of wheat to human diet and health. Food Energy Secur..

[15] Poole J.A., Barriga K., Leung D.Y., Hoffman M., Eisenbarth G.S., Rewers M., Norris J.M. (2006). Timing of Initial Exposure to Cereal Grains and the Risk of Wheat Allergy. Pediatrics.

[16] Venter C., Pereira B., Voigt K., Grundy J., Clayton C.B., Higgins B., Arshad S.H., Dean T. (2007). Original article: Prevalence and cumulative incidence of food hypersensitivity in the first 3 years of life. Allergy.

[17] Venter C., Pereira B., Grundy J., Clayton C.B., Arshad S.H., Dean T. (2006). Prevalence of sensitization reported and objectively assessed food hypersensitivity amongst six-year-old children: A population-based study. Pediatr. Allergy Immunol..

[18] Verrill L., Bruns R., Luccioli S. (2015). Prevalence of self-reported food allergy in U.S. adults: 2001, 2006, and 2010. Allergy and Asthma Proceedings.

[19] Vierk K.A., Koehler K.M., Fein S.B., Street D.A. (2007). Prevalence of self-reported food allergy in American adults and use of food labels. J. Allergy Clin. Immunol..

[20] Rance F., Grandmottet X., Grandjean H. (2005). Prevalence and main characteristics of schoolchildren diagnosed withfood allergies in France. Clin. Exp. Allergy.

[21] Woods R., Stoney R., Raven J., Walters E., Abramson M., Thien F. (2002). Reported adverse food reactions overestimate true food allergy in the community. Eur. J. Clin. Nutr..

[22] Pereira B., Venter C., Grundy J., Clayton C.B., Arshad S.H., Dean T. (2005). Prevalence of sensitization to food allergens, reported adverse reaction to foods, food avoidance, and food hypersensitivity among teenagers. J. Allergy Clin. Immunol..

[23] Ebisawa M., Ito K., Fujisawa T., Committee for Japanese Pediatric Guideline for Food Allergy, The Japanese Society of Pediatric Allergy and Clinical Immunology, The Japanese Society of Allergology (2020). Japanese guidelines for food allergy 2020. Allergol. Int..

[24] Gupta R.S., Springston E.E., Warrier M.R., Smith B., Kumar R., Pongracic J., Holl J.L. (2011). The Prevalence, Severity, and Distribution of Childhood Food Allergy in the United States. Pediatrics.

[25] Renz H., Allen K.J., Sicherer S.H., Sampson H.A., Lack G., Beyer K., Oettgen H.C. (2018). Food allergy. Nat. Rev. Dis. Prim..

[26] U.S. Food & Drug Administration (2022). Food Allergies: What You Need to Know. https://www.fda.gov/food/buy-store-serve-safe-food/food-allergies-what-you-need-know.

[27] Jin Y., Acharya H.G., Acharya D., Jorgensen R., Gao H., Secord J., Ng P.K.W., Gangur V. (2019). Advances in Molecular Mechanisms of Wheat Allergenicity in Animal Models: A Comprehensive Review. Molecules.

[28] Cianferoni A. (2016). Wheat allergy: Diagnosis and management. J. Asthma Allergy.

[29] Gao H., Jorgensen R., Raghunath R., Nagisetty S., Ng P.K.W., Gangur V. (2021). Creating hypo-/nonallergenic wheat products using processing methods: Fact or fiction?. Compr. Rev. Food Sci. Food Saf..

[30] Nakamura A., Tanabe S., Watanabe J., Makino T. (2005). Primary Screening of Relatively Less Allergenic Wheat Varieties. J. Nutr. Sci. Vitaminol..

[31] Kohno K., Takahashi H., Endo T.R., Matsuo H., Shiwaku K., Morita E. (2016). Characterization of a hypoallergenic wheat line lacking ω-5 gliadin. Allergol. Int..

[32] Larré C., Lupi R., Gombaud G., Brossard C., Branlard G., Moneret-Vautrin D., Rogniaux H., Denery-Papini S. (2011). Assessment of allergenicity of diploid and hexaploid wheat genotypes: Identification of allergens in the albumin/globulin fraction. J. Proteom..

[33] Gao H., Jorgensen R., Raghunath R., Ng P.K.W., Gangur V. (2022). An Adjuvant-Free Mouse Model Using Skin Sensitization Without Tape-Stripping Followed by Oral Elicitation of Anaphylaxis: A Novel Pre-Clinical Tool for Testing Intrinsic Wheat Allergenicity. Front. Allergy.

[34] Khodoun M.V., Strait R., Armstrong L., Yanase N., Finkelman F.D. (2011). Identification of markers that distinguish IgE- from IgG-mediated anaphylaxis. Proc. Natl. Acad. Sci. USA.

[35] Lewis J.M., Siler L., Souza E., Ng P.K.W., Dong Y., Brown-Guedira G., Jiang G.-L., Ward R.W. (2010). Registration of ‘Ambassador’ Wheat. J. Plant Regist..

[36] Sissons M. (2008). Role of Durum Wheat Composition on the Quality of Pasta and Bread. Food.

[37] Nagelkirk M., Black R. (2012). Wheat Varieties Used in Michiagn. https://www.canr.msu.edu/news/wheat_varieties_used_in_michigan.

[38] Hidalgo A., Brandolini A. (2014). Nutritional properties of einkorn wheat (*Triticum monococcum* L.). J. Sci. Food Agric..

[39] Shewry P.R. (2009). Wheat. J. Exp. Bot..

[40] Gonipeta B., Kim E., Gangur V. (2015). Mouse Models of Food Allergy: How Well do They Simulate the Human Disorder?. Crit. Rev. Food Sci. Nutr..

[41] Birmingham N.P., Parvataneni S., Hassan H.M.A., Harkema J., Samineni S., Navuluri L., Kelly C.J., Gangur V. (2007). An Adjuvant-Free Mouse Model of Tree Nut Allergy Using Hazelnut as a Model Tree Nut. Int. Arch. Allergy Immunol..

[42] Masthoff L.J., Hoff R., Verhoeckx K.C.M., Van Os-Medendorp H., Michelsen-Huisman A., Baumert J.L., Pasmans S.G., Meijer Y., Knulst A.C. (2013). A systematic review of the effect of thermal processing on the allergenicity of tree nuts. Allergy.

[43] Vanga S.K., Singh A., Raghavan V. (2017). Review of conventional and novel food processing methods on food allergens. Crit. Rev. Food Sci. Nutr..

